# An educational study to investigate the efficacy of three training methods for infiltration techniques on self-efficacy and skills of trainees in general practice

**DOI:** 10.1186/s12875-019-1023-7

**Published:** 2019-09-14

**Authors:** Nele R. Michels, Els Vanhomwegen

**Affiliations:** Centre for General Practice, Faculty of Medicine and Health Sciences, University of Antwerp, Doornstraat 331, 2610 Antwerp, Belgium

**Keywords:** Medical education, Musculoskeletal disorders, Injections, Intra-articular or periarticular, Models, Anatomic, Cadavers, Self-efficacy, Skills training

## Abstract

**Background:**

Research shows that few general practitioners perform intra- and periarticular infiltrations. Lack of good training strategies to teach these skills would be an important reason for this observation. In this study, we investigated and compared three different training strategies for infiltrations of the glenohumeral joint, subacromial space, lateral epicondyle, carpal tunnel and knee joint.

**Methods:**

Trainees in general practice were randomized into three teaching groups: a theoretical lecture (*n* = 18), or a theoretical lecture with training on anatomical models (*n* = 19) or with a training on cadavers (*n* = 11). The study period was 3 months. Before and after the training, the self-efficacy (questionnaire) and skills (Objective Structured Clinical Examination or OSCE, test on anatomical models) were evaluated. The self-efficacy was assessed again 3 months later. A Kruskal-Wallis test was used to compare the results before versus after training and between groups (*p* < 0.05).

**Results:**

All three training strategies had a significantly positive effect on the self-efficacy concerning knowledge and skills. This benefit remained 3 months after training. However, some participants still felt uncomfortable to perform infiltrations. Best scores for self-efficacy concerning skills and best scores on the OSCE were observed after training on cadavers, followed by training on anatomical models.

**Conclusions:**

Based on this study we suggest the combination of a theoretical lecture with a training on cadavers to teach infiltration techniques. To achieve an optimal long-term effect, additional refresher trainings may be necessary.

## Background

Musculoskeletal diseases are frequent in general practice or family medicine, accounting for 20% of all consultations [1, 2]. For some of these diseases an intra- or periarticular infiltration can be a safe and effective treatment. Former studies and reviews showed that when physicians are trained and patients are selected properly, complications and adverse effects of the medication used are rather rare [3–5]. Furthermore, aspiration of intra-articular fluid can help in diagnosing musculoskeletal conditions like septic arthritis or gout [6]. Regions that are most frequently infiltrated in general practice are the subacromial region, the knee joint, the glenohumeral joint, the lateral epicondyle and the carpal tunnel [2]. Research shows that only a minority of general practitioners performs intra- and periarticular infiltrations. Insufficient experience and a lack of good strategies to train these skills could be an important reason for this observation [2, 6–9]. Often this kind of procedural skills are trained by the traditional method of apprenticeship, where trainees perform the procedure on patients after a demonstration by the trainer or supervisor at the workplace. At some institutions infiltration techniques are taught in skills labs during undergraduate or postgraduate courses, although often these courses are voluntary.

Previous studies could already demonstrate the beneficial effect of a theoretical training or a training on patients, anatomical models or cadavers on comfort level or self-efficacy, knowledge and/or skills for infiltration techniques [1, 8, 10]. Sterrett and colleagues (2011) demonstrated an improvement in comfort level by a sufficient number of students and residents after a training with anatomical models [10]. Kay et al. (2016) only studied the effect of a training on cadavers. They found overall statistically significant improvements in both comfort levels and skills when comparing pre- and post-tests [8]. Some studies also compared different training methods. Gormley et al. (2003) demonstrated that a training on patients in addition with training on anatomical models of the shoulder joint results in a higher increase of confidence and number of infiltrations performed in practice compared with a training on anatomical models alone [11]. Four studies compared a theoretical lecture with a training on anatomical models of the shoulder and knee joint, concluding that the latter has a greater impact on confidence, knowledge and skills for infiltrations and on the number of infiltrations performed in practice [12–15]. One small study (*n* = 7) demonstrated that participants in a training on cadavers had higher comfort scores for infiltrations compared with a training on anatomical models. Because of the small sample size, the results of this study must be interpreted with caution [16].

As more students, general practice trainees and general practitioners themselves should be trained in infiltration techniques, we should know which educational strategies to teach infiltration techniques give the best results. Subsequently, an evidence-based advice to teaching organizations could be formulated. We set up an educational randomized controlled trial with the following research question: Which training strategies are the most effective to teach infiltration techniques of the five most frequently infiltrated anatomical regions? Therefore we investigated and compared the impact of using only theoretical lectures or a combination of a theoretical lecture with two other educational methods, i.e. a training on anatomical models and a training on cadavers. Furthermore, we chose to investigate teaching in infiltration techniques of the five most frequently infiltrated anatomical regions: the glenohumeral joint, the subacromial region, the lateral epicondyle, the carpal tunnel and the knee joint. As outcomes we measured self-efficacy and skills. Self-efficacy is defined by Bandura as people’s beliefs in their capabilities to produce given attainments [17].

## Methods

### The Flemish postgraduate general practice curriculum

In Flanders (Belgium), four academic centres (University of Antwerp, Vrije Universiteit Brussel, Ghent University and KU Leuven) and the Interuniversity Centre for General Practice (ICHO) organise a three-year postgraduate general practice training. The training is workplace based, which means that general practice trainees work independently most of the time both in a general practice (2,5 years) and at a hospital ward (0,5 year). This means that they see and visit patients on their own. Trainees are directly coached by a trainer at the practice and receive extra tutoring by a practice-coordinator. Next to the work at the practice, some time for academic activities is reserved. Once a week, trainees follow peer tutoring or intervision sessions, obliged courses and/or elective courses and they can study or spent some time at their master thesis.

### Study protocol

**Fig. 1:**
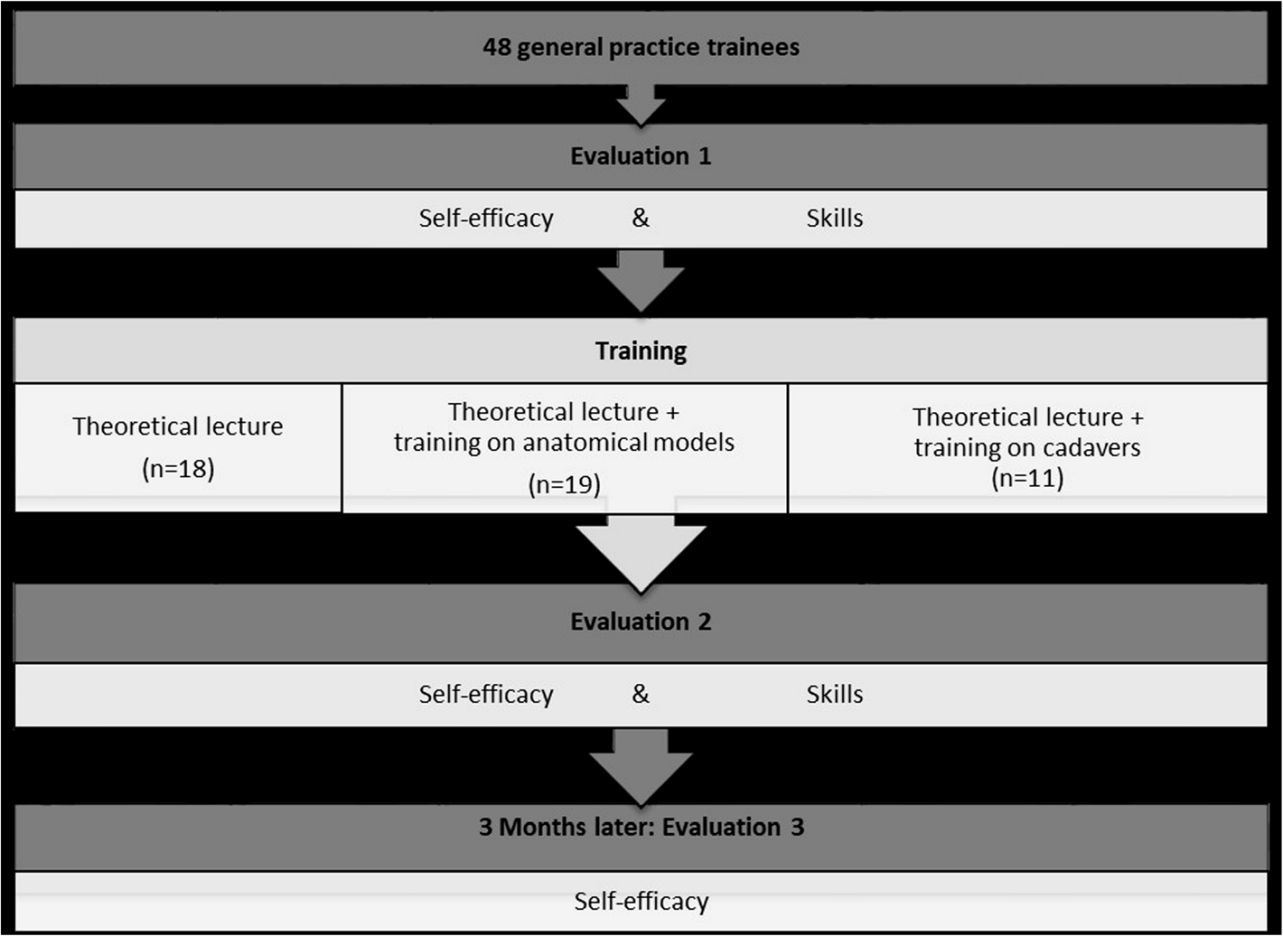
Study design. The total study period was 3 months. Evaluation 1, training and evaluation 2 were done on the same day. Evaluation 3 was carried out 3 months later. For training, the 48 participants were divided into 3 groups: a theoretical lecture, a lecture with a training on anatomical models and a lecture with training on cadavers

The study design is demonstrated in Fig. 1. General practice trainees in Flanders (Belgium) were recruited by e-mail and social media in October–November 2015 for an elective course ‘joint infiltrations’. Trainees were aware to be included in the study and based on their characteristics (university, training year and sex), they were randomized into three training groups. Before the start of the study all participants received an information letter and they were asked to fill in the informed consent. The study was approved by the ethical committee of the University Hospital of Antwerp (registration No B300201525035). Before and after the course the participants’ self-efficacy and skills were evaluated (see infra). The self-efficacy was assessed again 3 months later with an online survey.

### Training strategies for joint infiltrations

Three different courses were organized for three different trainee groups: a theoretical lecture, a theoretical lecture combined with a training on anatomical models and a theoretical lecture combined with a training on cadavers. In the theoretical lecture, infiltration techniques of five anatomical regions were taught: the glenohumeral joint, the subacromial region, the lateral epicondyle, the carpal tunnel and the knee joint. All participants were given a PowerPoint presentation they could peruse at their own pace. The content of this presentation was based on guidelines of infiltration techniques for general practitioners [3, 18–22] and complemented with instruction videos, recorded in the practice of the researchers. Next to instructions on performance also information on possible complications of joint infiltration and informed consent of patients were given; special attention was given to communication and informing patients and to essential steps as regards sterility and hygiene procedures. After this lecture, the first trainee group did not received any further training. The second trainee group had the opportunity to practice on anatomical models, a third trainee group could train on cadavers. Trainings on anatomical models and cadavers were supervised by general practitioners experienced in infiltration techniques. The anatomical models had a build-in visual feedback system (Limbs & Things™, product numbers 30,031, 30,080, 30,010 and 70,020) (Images of the models are added, see Additional file 1). As regards the cadavers, a fixated corpse in prone position was used for infiltrations of the glenohumeral joint, the subacromial region, the lateral epicondyle and the carpal tunnel. To practice infiltrations of the knee joint, a fresh-frozen leg was provided. The unembalmed Caucasian cadavers were donated to the Laboratory of Human Anatomy and Embryology of the Faculty of Medicine and Health Sciences, University of Antwerp, by means of a written testament to being used after death for scientific and educational purposes.

### Evaluation

Self-efficacy was evaluated using a questionnaire (see Additional file 2) in which participants were asked to estimate their own perceived knowledge-level, motivation and skills as regards infiltration techniques of the five anatomical regions. This questionnaire was based on the guidelines of Bandura [17]. The answers on this questionnaire were converted into an ordinal Likert-scale (from strongly disagree = 0 to strongly agree = 5).

To evaluate the infiltration skills, participants were asked to perform infiltrations of the five anatomical regions on anatomical models. They were evaluated in a not blinded but objective, structured way (Objective Structured Clinical Examination (OSCE)) [23]. Participants needed to circulate through 6 stations: one station for each type of joint-infiltration: the glenohumeral joint, the subacromial region, the lateral epicondyle, the carpal tunnel and the knee joint lateral approach and anterior approach. Time foreseen per station was 7 min. Participants were evaluated on their skills to perform the infiltration conform seven important steps for infiltrations, i.e. the assessment criteria: positioning, palpation, identification of the injection site, disinfection and sterility, needle position, aspiration and injection, informing the patient). Each correctly performed step counted for one point. On a more global rating scale the trainees’ proficiency (the fluency of acting), systematic (logical order of the different steps) and completeness (performance of all the steps) of performing the skills were evaluated with a score ranging from 0 to 10 [24]. The assessors were general practitioners experienced in infiltration techniques.

### Analysis

The median, minimal and maximal scores of both the self-efficacy evaluation and the seven steps of the OSCE were calculated. Results of the trainees’ proficiency, systematics and completeness at the OSCE were calculated as a mean and standard deviation. Statistical analysis was carried out using IBM SPSS Statistics 23 for Windows. A Kruskal-Wallis test was used to compare the participants’ self-efficacy and skills before versus after training and between groups (*p* < 0.05).

## Results

### Participants

About 560 general practice trainees were invited for the elective course ‘joint infiltrations’, 48 (8.6%) of them registered and thus participated in the study from November 2015 to January 2016. We recruited more female (*n* = 40, 83.3%) than male (*n* = 8) trainees. We had a realistic spread over the 4 Flemish training institutions (Antwerp (29.2%), Brussels (4.2%), Ghent (14.6%), and Leuven (52.1%)), taking into account the numbers of trainees per university. 54.2% of the participants were first year trainees, 45.8% second year trainees. All participants were equally divided among the 3 different training groups, considering training institution, sex and year of training.

Eighteen trainees (37.5%) participated in the first training group with only a theoretical lecture, 19 trainees (39.6%) participated in the second training group (theoretical lecture with training on anatomical models) and 11 trainees (22.9%) in the third training group (theoretical lecture with training on cadavers).

### Self-efficacy

#### Comparison before versus after training (pre, post & 3 months post)

All three training strategies had a significantly positive effect on the participants’ self-efficacy concerning knowledge level and skills (see Table 1). Immediately after the training, the knowledge level scores increased from ‘somewhat disagree’ and ‘somewhat agree’ to ‘agree’ or ‘strongly agree’. This beneficial effect remained 3 months after the course. Although some scores slightly decreased again after 3 months, these decreases were not significant. The scores (minimal-maximal value) also show that some participants still felt uncomfortable to perform infiltrations after training (scores of 2 (somewhat disagree) or lower), and this for all the training groups and for all kind of infiltrations. The perceived motivation to learn infiltration techniques was already high and did not change significantly immediately and 3 months after the training.

**Table 1:** Results of the self-efficacy questionnaire

	median									minimal-maximal value								
	pre			post			3 months post			pre			post			3 months post		
	G1	G2	G3	G1	G2	G3	G1	G2	G3	G1	G2	G3	G1	G2	G3	G1	G2	G3
knowledge-level																		
gleno-humeral	3	2	3	4	4	5	4	4	4	0–4	0–4	0–4	3–5	3–5	3–5	2–4	3–5	2–5
sub-acromial	3	3	2	4	4	4	4	4	4	1–5	0–4	0–4	3–5	3–5	3–5	2–5	2–5	2–5
lateral epic.	2.5	2	2	5	4	4	4	4	4	1–5	0–3	0–3	3–5	4–5	3–5	2–5	2–5	2–5
carpal tunnel	2	3	3	4	4	4	4	4	4	0–5	0–5	0–4	3–5	4–5	3–5	1–5	3–5	3–5
knee	3	3	3	4	4	5	4	3	4	1–5	0–4	1–4	3–5	3–5	3–5	3–5	2–5	3–5
motivation																		
gleno-humeral	5	5	5	5	5	5	5	4	4.5	3–5	1–5	4–5	4–5	3–5	4–5	3–5	4–5	2–5
sub-acromial	5	5	5	5	5	5	5	5	4.5	3–5	1–5	4–5	3–5	3–5	4–5	4–5	4–5	3–5
lateral epic.	4	4	5	5	4	4	4	4	4	0–5	3–5	0–5	2–5	3–5	1–5	2–5	2–5	1–5
carpal tunnel	4.5	4	5	5	4	5	4	4	5	2–5	2–5	4–5	3–5	3–5	3–5	2–5	2–5	3–5
knee	5	5	5	5	5	5	5	5	5	2–5	1–5	3–5	3–5	2–5	4–5	4–5	3–5	4–5
skills																		
gleno-humeral	2	1	1	**4**	**4**	**4**	**3**	**3**	**3.5**	0–4	0–3	0–4	**3–4**	**3–5**	**3–5**	**2–5**	**1–5**	**2–5**
sub-acromial	2	2	1	**4**	**4**	**4**	**4**	**3**	**4**	0–5	0–4	0–3	**3–5**	**3–5**	**3–5**	**2–5**	**1–5**	**2–5**
lateral epic.	1	1	1	**4**	**4**	**4**	**4**	**4**	**4**	0–4	0–3	0–4	**3–5**	**2–5**	**1–5**	**2–5**	**1–5**	**2–5**
carpal tunnel	1	2	1	**3**	***4***	**4**	**3**	**3**	**4**	0–5	0–4	0–4	**2–5**	***1–5***	**3–5**	**0–4**	**1–5**	**2–5**
knee	2.5	1	2	**4**	**3**	**4**	**4**	**3**	**4**	0–4	0–3	0–4	**2–5**	**1–5**	**4–5**	**2–5**	**1–4**	**3–5**

Results are given as median and minimal – maximal value

0 = strongly disagree, 1 = disagree, 2 = somewhat disagree, 3 = somewhat agree, 4 = agree, 5 = strongly agree

pre = value before training, post = value immediately after training, 3 months post = value 3 months after training

*G1* group 1: only theoretical lecture, *G2* group 2: theoretical lecture + anatomic models, *G3* group 3: theoretic lecture + cadavers

**Scores in bold**: scores post training are significantly higher than before training (*p* ≤ 0,05)

***Scores in italic*:** score (carpal tunnel)(G2) is significantly higher than after theoretical lecture (G1) (*p* ≤ 0,05)

Scores with a full underline: Scores (knee)(G1 & G3) are significantly higher than after lecture + training on anatomical models (G2) (*p* ≤ 0,05)

#### Comparison between the three training strategies

No significant differences in perceived knowledge level and motivation were found (see Table 1). Participants in the training on cadavers estimated their skills for infiltrations of the knee joint significantly higher than participants in the training on anatomical models. This effect was observed immediately after and 3 months after training. Participants in the training on anatomical models estimated their skills for infiltrations of the carpal tunnel significantly higher than participants in the group of theoretical lecture only. This effect was only present immediately after training. Participants in the theoretical lecture-group and the training on cadavers estimated their skills for infiltrations of the knee joint significantly higher than participants in the training on anatomical models. This observation was present immediately after training and 3 months after training.

### Skills

To evaluate the progress of the participants’ skills a skills test or OSCE before and after the training was organized. Assessment criteria were the number of correctly performed steps and if the performance was proficient, systematic and complete. Results of the former are given in Table 2, of the latter in Table 3.

**Table 2:** number of correctly performed steps for infiltrations on the skills test or OSCE

	OSCE											
	median						minimal-maximal value					
	pre			post			pre			post		
	G1	G2	G3	G1	G2	G3	G1	G2	G3	G1	G2	G3
gleno-humeral	1.5	3	3	**6**	***7***	***7***	0–5	0–6	0–5	**5–7**	***5–7***	***6–7***
sub-acromial	4	3	5	**6**	***7***	***7***	0–7	0–7	3–7	**5–7**	***6–7***	***7–7***
lateral epic.	4	4	4	**7**	**6**	**7**	1–7	0–6	0–6	**3–7**	**3–7**	**6–7**
carpal tunnel	4	4	4	**6**	***7***	***7***	1–7	2–6	2–7	**5–7**	***5–7***	***7–7***
knee LA	4	5	4	**7**	**7**	**7**	3–7	2–7	0–7	**5–7**	**6–7**	**7–7**
knee AA	3	3	1	**7**	**7**	**7**	0–7	0–6	0–7	**6–7**	**5–7**	**7–7**

Results are given as median and minimal – maximal value

pre = value before training, post = value immediately after training

*G1* group 1: only theoretical lecture, *G2* group 2: theoretical lecture + anatomic models, *G3* group 3: theoretic lecture + cadavers

Knee *AA* anterior approach, *LA* lateral approach

**Scores in bold**: scores post training are significantly higher than before training (p ≤ 0,05)

***Scores in italic*:** scores (G2 & G3) are significantly higher than after theoretical lecture (G1) (p ≤ 0,05)

Scores with a full underline: Scores (G3) are significantly higher than after lecture + training on anatomical models (G2) (*p* ≤ 0,05)

**Table 3:** OSCE results on proficiency, systematic and completeness

	pre						post					
	G1		G2		G3		G1		G2		G3	
	m	SD	m	SD	m	SD	m	SD	m	SD	m	SD
proficiency												
gleno-humeral	4,3	±1.7	4,5	±2.0	3,4	±2.0	**7.3**	**±0.8**	***8.3***	***±0.9***	**9.2**	**±1.0**
sub-acromial	4.4	±2.3	3.7	±2.6	3.6	±1.3	**7.2**	**±0.7**	***8.2***	***±0.7***	**9.1**	**±0.9**
lateral epic.	4.7	±1.4	3.3	±1.4	3.3	±1.7	**7.6**	**±0.8**	**7.7**	**±1.0**	**9.1**	**±0.9**
carpal tunnel	3.8	±1.4	4.1	±1.7	5.2	±1.8	**7.2**	**±0.9**	***8.0***	***±1.0***	***8.4***	***±0.7***
knee LA	4.9	±1.8	5.1	±1.7	3.9	±2.5	**7.7**	**±0.8**	**7.8**	**±0.9**	**7.8**	**±0.8**
knee AA	3.3	±2.0	3.6	±2.6	2.5	±3.1	**7.4**	**±0.9**	**8.1**	**±0.9**	**7.7**	**±0.8**
systematics												
gleno-humeral	4,3	±1.8	4,5	±2.1	3,7	±2.0	**7.5**	**±0.9**	***8.4***	***±0.8***	**9.4**	**±0.7**
sub-acromial	4.5	±2.4	3.6	±2.8	3.9	±1.1	**7.5**	**±0.7**	***8.3***	***±0.7***	**9.5**	**±0.7**
lateral epic.	5.4	±1.1	4.2	±1.9	3.5	±1.8	**7.6**	**±0.9**	***8.3***	***±0.7***	**9.5**	**±0.7**
carpal tunnel	4.3	±1.5	4.8	±1.4	5.3	±1.8	**7.8**	**±0.6**	**8.2**	**±0.8**	***8.5***	***±0.5***
knee LA	5.0	±1.8	5.4	±1.6	3.6	±2.5	**7.7**	**±0.7**	**8.1**	**±0.7**	**8.1**	**±0.5**
knee AA	3.4	±2.2	3.7	±2.8	2.3	±3.0	**7.7**	**±0.6**	***8.3***	***±0.7***	**8.0**	**±0.6**
completeness												
gleno-humeral	4,2	±0.8	4,4	±2.3	3,8	±2.1	**8.0**	**±0.8**	**8.4**	**±0.6**	**9.5**	**±0.7**
sub-acromial	4.7	±2.4	3.6	±2.8	4.1	±1.3	**8.0**	**±0.8**	**8.4**	**±0.7**	**9.5**	**±0.7**
lateral epic.	5.2	±1.2	4.3	±1.8	3.7	±1.9	**8.0**	**±1.0**	**8.4**	**±0.6**	**9.5**	**±0.7**
carpal tunnel	4.2	±1.6	4.8	±1.5	5.2	±1.7	**7.8**	**±0.6**	***8.3***	***±0.7***	**8.3**	**±0.6**
knee LA	4.7	±1.9	5.3	±1.8	4.1	±2.7	**8.1**	**±0.5**	**8.3**	**±0.7**	**8.1**	**±0.7**
knee AA	3.3	±2.2	3.7	±2.9	2.5	±3.3	**7.8**	**±0.6**	***8.4***	***±0.8***	**8.3**	**±0.6**

Results are given as median (m) and standard deviation (SD)

pre = value before training, post = value immediately after training

*G1* group 1: only theoretical lecture, *G2* group 2: theoretical lecture + anatomic models, *G3* group 3: theoretic lecture + cadavers

Knee *AA* anterior approach, *LA* lateral approach

**Scores in bold**: scores post training are significantly higher than before training (p ≤ 0,05)

***Scores in italic*:** scores (G2 & G3) are significantly higher than after theoretical lecture (G1) (*p* ≤ 0,05)

Scores with a full underline: Scores (G3) are significantly higher than after lecture + training on anatomical models (G2) (*p* ≤ 0,05)

#### Comparison before versus after training (pre & post)

As can be seen in Tables 2 and 3, the participants’ performances significantly improved and this independently of the training strategy that was used. Participants showed to be better in performing all the different steps belonging to infiltrations and this with a higher proficiency, with a better systematic and more complete.

#### Comparison between the three training strategies

For infiltrations of the glenohumeral joint, the subacromial region and the carpal tunnel participants who followed the training on anatomical models or cadavers significantly performed more steps in a correct way than participants who followed only the theoretical lecture. For infiltrations of the lateral epicondyle and the knee joint (anterior approach) participants who followed the training on cadavers significantly performed more steps in a correct way than participants who followed the theoretical lecture only or the training on anatomical models.

Compared with participants in the theoretical lecture, participants in the training on anatomical models significantly performed infiltrations more proficient as regards the glenohumeral joint, the subacromial region, and the carpal tunnel. They worked significantly more systematically for infiltrations of the glenohumeral joint, the subacromial region, the lateral epicondyle and the knee joint through anterior approach. The carpal tunnel- and knee joint (anterior approach)-infiltrations were significantly performed more complete.

Compared with participants in the theoretical lecture or training on anatomical models, participants in the training on cadavers had significantly higher scores on the OSCE for proficiency, systematics and completeness when infiltrating the glenohumeral joint, the subacromial space and the lateral epicondyle. Moreover, these participants had significantly higher scores for proficiency and systematics when infiltrating the carpal tunnel compared with participants in the group of the theoretical lecture only.

## Discussion

In this study we dealt with the research question: Which training strategies are the most effective to teach infiltration techniques of the five most frequently infiltrated anatomical regions? As general practitioners often need to diagnose and treat patients with musculoskeletal problems, it is certainly relevant to educate general practice trainees and general practitioners in these techniques. Based on this study we can advise to teaching organizations that all studied training strategies, a theoretical lecture only or combined with training on anatomical models or training on cadavers, are effective. In addition, our results demonstrate that the combination of theoretical knowledge with a practical training, especially with a training on cadavers, reveal better results on skill performance. Although, not always and not for all kind of infiltrations. As regards a more proficient, systematic and complete performance, this was the case for infiltrations of the gleno-humeral and the sub-acromial space and the lateral epicondyle but not for the carpal tunnel and the knee joint (both lateral and anterior approach); for the latter the use of anatomical models seemed to work better. As regards the correct performance of all relevant steps when infiltrating, we only saw a greater value of cadaver training for lateral epicondyle and anterior approach of the knee joint.

Next to objective measures of skill performance, we also evaluated how confident (self-efficacy) trainees felt before and after training to perform infiltrations. We repeated this evaluation also 3 months after the training. Results are less clear to interpret. Overall, no clear differences between the training strategies were found and confidence in knowledge level and skills improved even 3 months later. There was no change in the perceived motivation but participants were already highly motivated to perform infiltrations from the beginning. Furthermore, we observed some discordance between the self-efficacy of the participants and their observed skills. For example, after the theoretical lecture participants estimated their own skills for infiltrations of the knee higher than after training on anatomical models, while the opposite was observed in the skills test (see Table 2). Other studies confirm there is only little association between the self-assessments of medical doctors and scores on a skill test [25]. Finally, we want to stress that the measured minimal and maximal values (Table 1) demonstrate that a small proportion of participants still feel uncomfortable to perform infiltrations in vivo after training. This means that, nevertheless good first trainings, refreshing courses or an extra-guided training on patients might be necessary.

The fact that the achievement of procedural skills improves by a combination of practical training with theoretical training, seems obvious and is also supported by the results of many other studies, as well as in general educational studies [26] as for infiltrations in particular [11–16, 27]. Moreover, these former studies on infiltration techniques also concluded that training on cadavers seemed to be the most effective, followed by training on anatomical models. As regards repeated and refreshing trainings and a training at the workplace, educational theories support this approach [26, 28]; we did not find data in literature, however, about content and frequency of additional specific infiltrations training. At the workplace, sufficient and effective guiding from trainers and supervisors is essential [29, 30].

The strength of this study is the comparison of three different training methods to teach infiltration techniques on five different anatomical regions. We measured not only skills in an objective way but also perceived performances. Besides, a relative basic sample size was used and, to evaluate the effect of training on a longer term, we continued this study for 3 months. Former studies on training methods of infiltration techniques were rather small [16] or compared only two training methods [11–14, 16]. Although infiltrations of the lateral epicondyle and carpal tunnel are frequently carried out in general practice [2], most studies included only training of infiltrations of the knee and/or shoulder joint [11–13, 15].

We acknowledge some limitations of this study. First, we realize that for some conclusions the sample size was still too small. The training in joint infiltrations was offered as an elective course. As elective courses have a mean participation rate of 30 trainees, we can say that this elective course was rather popular among trainees. Secondly, for practical reasons both participants and observers at the skills test were not blinded. Consequently, the results and skills tests can be biased. Finally, the costs and feasibility of the three training strategies were not considered in this study. A theoretical lecture is of course the most inexpensive and feasible method. Once the lecture is implemented, trainees can peruse it, even in an online course. Both in case of training on anatomical models and cadavers the surveillance and assistance of an experienced physician is needed. Furthermore, the costs of the anatomical models and the cadavers must be considered. In this study, four anatomical models were needed with a cost of about 2000 euro each. These models can of course be reused for several trainings. For the training on cadavers, two corpses were needed, one in prone and one in supine position. To prepare a cadaver for training, about 100 euro for material is needed. After training in infiltration techniques, cadavers can be used for other trainings, for example a surgical training, but the durability is limited. The major restriction to use cadavers to teach infiltration techniques might be the shortage of bodies that are donated to scientific research.

### Implications

Based on this study, we can suggest a combination of a theoretical lecture and training on cadavers or anatomical models to teach infiltration techniques. Probably refreshing courses, both on models and in vivo are also valuable. Our suggestions for further research on this topic are: [1] conduction of quality studies with blinding of observers and larger sample sizes to exclude bias of the study results; and [2] determination of the type and frequency of refreshing trainings.

## Conclusion

Both a lecture on infiltration techniques and a combination of a lecture with training on anatomical models or cadavers have a beneficial effect on the self-efficacy and skills of trainees in general practice. However, best results on skills seem to be achieved after training on cadavers, followed by training on anatomical models. In addition to the efficacy of these training strategies, the preferred method in which infiltration techniques are taught will also depend on costs and practical feasibility. Furthermore refreshing courses or guided training on patients might be necessary before trainees can perform infiltrations independently on patients.

## Supplementary information

Additional file 1. Images of the anatomical models used. Images of the different anatomical models that were used for participants in the second training group; they had the opportunity to practice on anatomical models with a build-in visual feedback system. (DOCX 204 kb)

Additional file 2. Self-efficacy questionnaire to estimate perceived knowledge-level, motivation and skills as regards infiltration techniques. Self-efficacy was evaluated using a questionnaire in which participants were asked to estimate their own perceived knowledge-level, motivation and skills as regards infiltration techniques of the five anatomical regions. This questionnaire was based on the guidelines of Bandura (2006). The answers on this questionnaire were converted into an ordinal Likert-scale (from strongly disagree ‘---‘ = 0 to strongly agree ‘+++’ = 5). (DOCX 37 kb)

## Data Availability

The datasets used and/or analysed during the current study are available from the corresponding author on reasonable request.

## Abbreviations

ICHO: Interuniversity Centre for General Practice
OSCE: Objective Structured Clinical Examination
