# Supplementary material for: An educational study to investigate the efficacy of three training methods for infiltration techniques on self-efficacy and skills of trainees in general practice
Source: BMC Fam Pract. 2019 Sep 14;20:133. doi: 10.1186/s12875-019-1023-7 (PMC6744665; doi:10.1186/s12875-019-1023-7)
Supplement: Supplementary file 1 — Images of the anatomical models used. Images of the different anatomical models that were used for participants in the second training group; they had the opportunity to practice on anatomical models with a build-in visual feedback system. (DOCX 204 kb) [file 12875_2019_1023_MOESM1_ESM.docx]

Additional file 1: Images of the anatomical models used

**Wrist**

Limbs & Things ^TM^, product number 30031


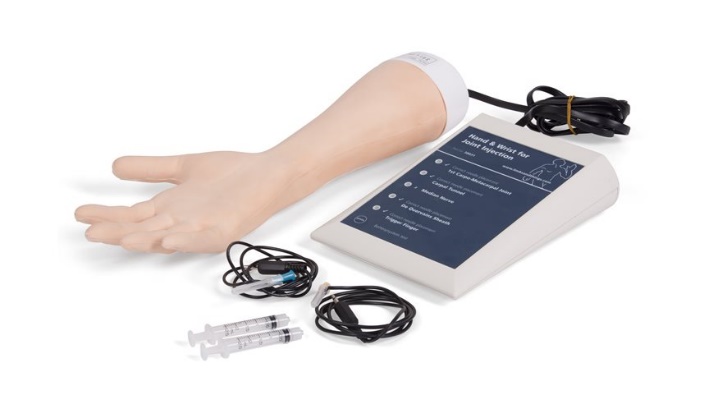

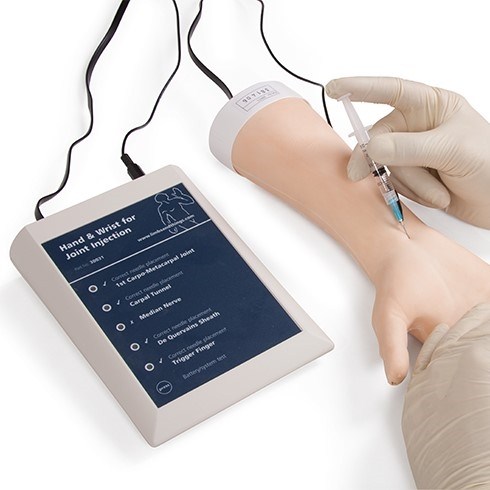


**Elbow**

Limbs & Things ^TM^, product number 30080


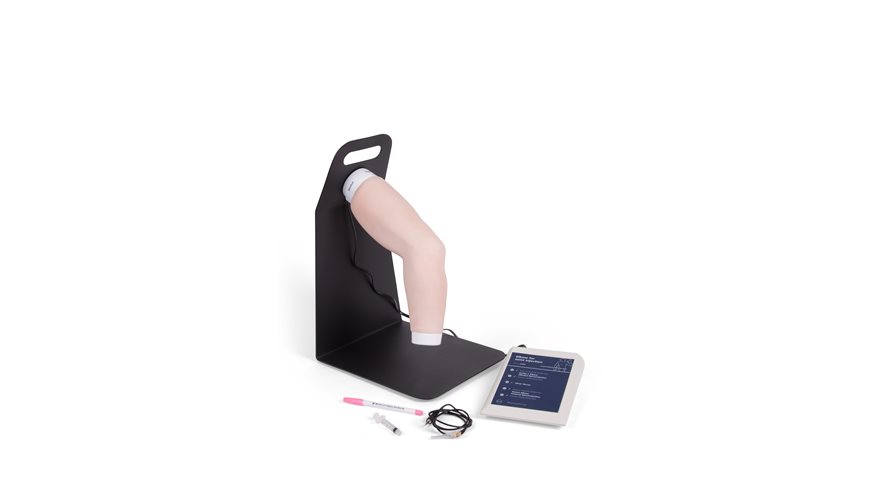


**Shoulder**

Limbs & Things ^TM^: product number 30010


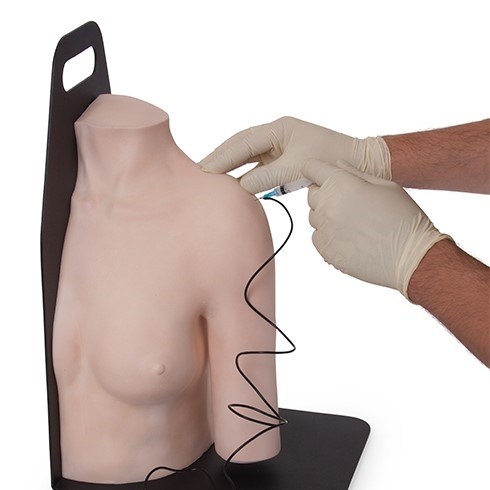

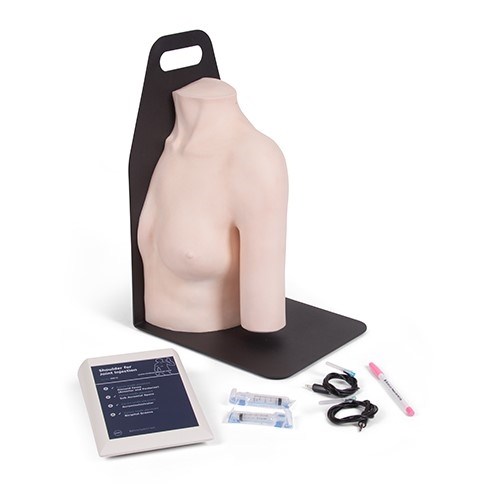


**Knee**

Limbs & Things ^TM^: product number 70020


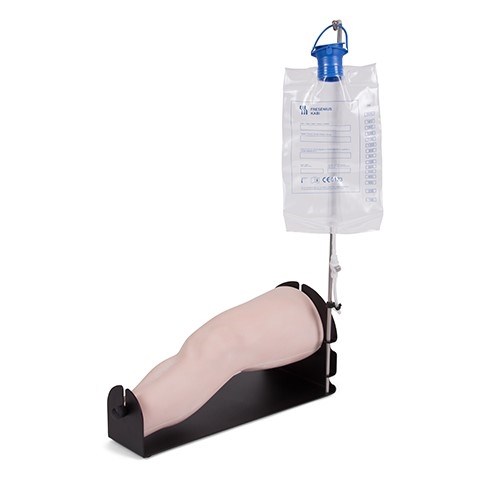

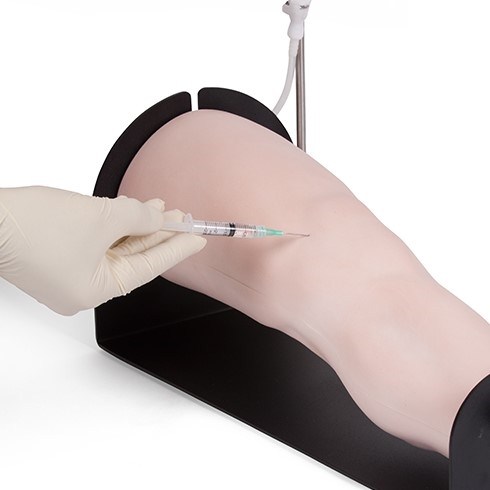


Permission to publish the images was received.
