# Supplementary material for: An educational study to investigate the efficacy of three training methods for infiltration techniques on self-efficacy and skills of trainees in general practice
Source: BMC Fam Pract. 2019 Sep 14;20:133. doi: 10.1186/s12875-019-1023-7 (PMC6744665; doi:10.1186/s12875-019-1023-7)
Supplement: Supplementary file 2 — Self-efficacy questionnaire to estimate perceived knowledge-level, motivation and skills as regards infiltration techniques. Self-efficacy was evaluated using a questionnaire in which participants were asked to estimate their own perceived knowledge-level, motivation and skills as regards infiltration techniques of the five anatomical regions. This questionnaire was based on the guidelines of Bandura (2006). The answers on this questionnaire were converted into an ordinal Likert-scale (from strongly disagree ‘---‘ = 0 to strongly agree ‘+++’ = 5). (DOCX 37 kb) [file 12875_2019_1023_MOESM2_ESM.docx]

## Additional file 2: Self-efficacy questionnaire to estimate perceived knowledge-level, motivation and skills as regards infiltration techniques

|  | I have theoretical knowledge about the skill,  🡪 I know this. | | | | | | I think this is an important skill,  🡪 I want this. | | | | | | I feel competent to perform this skill,  🡪 I can do this. | | | | | |
| --- | --- | --- | --- | --- | --- | --- | --- | --- | --- | --- | --- | --- | --- | --- | --- | --- | --- | --- |
|  | Strongly disagree | Disagree | Tend to disagre | Tend to agree | Agree | Strongly agree | Strongly disagree | Disagree | Tend to disagre | Tend to agree | Agree | Strongly agree | Strongly disagree | Disagree | Tend to disagre | Tend to agree | Agree | Strongly agree |
|  |  | | | | | |  | | | | | |  | | | | | |
| Glenohumeral joint | - - - | - - | - | + | ++ | +++ | - - - | - - | - | + | ++ | +++ | - - - | - - | - | + | ++ | +++ |
| Subacromial region | - - - | - - | - | + | ++ | +++ | - - - | - - | - | + | ++ | +++ | - - - | - - | - | + | ++ | +++ |
| Lateral epicondyle | - - - | - - | - | + | ++ | +++ | - - - | - - | - | + | ++ | +++ | - - - | - - | - | + | ++ | +++ |
| Carpal tunnel | - - - | - - | - | + | ++ | +++ | - - - | - - | - | + | ++ | +++ | - - - | - - | - | + | ++ | +++ |
| Knee joint | - - - | - - | - | + | ++ | +++ | - - - | - - | - | + | ++ | +++ | - - - | - - | - | + | ++ | +++ |
